# Comparison of Droplet Digital PCR and Quantitative PCR Assays for Quantitative Detection of *Xanthomonas citri* Subsp. *citri*

**DOI:** 10.1371/journal.pone.0159004

**Published:** 2016-07-18

**Authors:** Yun Zhao, Qingyan Xia, Youping Yin, Zhongkang Wang

**Affiliations:** School of Life Science, Chongqing University, Chongqing, 400030, China; Shanghai Jiao Tong University, CHINA

## Abstract

Droplet digital polymerase chain reaction (ddPCR) is a novel molecular biology technique providing absolute quantification of target nucleic acids without the need for an external calibrator. Despite its emerging applications in medical diagnosis, there are few reports of its use for the detection of plant pathogens. This work was designed to assess the diagnosis potential of the ddPCR for absolute quantitative detection of *Xanthomonas citri* subsp. *citri*, a quarantine plant pathogenic bacterium that causes citrus bacterial canker in susceptible *Citrus* species. We transferred an established quantitative PCR (qPCR) assay for citrus bacterial canker diagnosis directly to the ddPCR format and compared the performance of the two methods. The qPCR assay has a broader dynamic range compared to the ddPCR assay and the ddPCR assay has a significantly higher degree of sensitivity compared to the qPCR assay. The influence of PCR inhibitors can be reduced considerably in the ddPCR assay because the collection of end-point fluorescent signals and the counting of binomial events (positive or negative droplets) are associated with a Poisson algorithm. The ddPCR assay also shows lower coefficient of variation compared to the qPCR assay especially in low target concentration. The linear association of the measurements by ddPCR and qPCR assays is strong (Pearson correlation = 0.8633; *P*<0.001). Receiver operating characteristic analysis indicates the ddPCR methodology is a more robust approach for diagnosis of citrus bacterial canker. In summary, the results demonstrated that the ddPCR assay has the potential for the quantitative detection of *X*. *citri* subsp. *citri* with high precision and accuracy as compared with the results from qPCR assay. Further studies are required to evaluate and validate the value of ddPCR technology in the diagnosis of plant disease and quarantine applications.

## Introduction

Citrus bacterial canker (CBC) is a disease caused by the phytopathogens *Xanthomonas citri* subsp. *citri* (*Xcc*) a Gram-negative bacterium causing economically important diseases of citrus trees[1,2]. Any method designed to control and treat CBC effectively requires early identification of the causal pathogen. Serological and molecular assays have been developed to identify *Xcc* for diagnostic purposes[3–5]. Among them, quantitative polymerase chain reaction (qPCR) technology offers sensitive, specific and closed-tube detection, and identification of *Xcc* as well as other plant pathogens. A variety of qPCR-based assays are available[4–8] but in routine diagnosis or quarantine works, the results of most qPCR tests used for the detection of *Xcc* are usually interpreted qualitatively to show only *Xcc* positive or negative. Quantitative interpretation of qPCR assay results for *Xcc* as well as others plant pathogens, however, contains useful and meaningful information that is valuable for research, including epidemiological studies, bacterial infection kinetics, determination of biologically relevant threshold, and especially for screening cultivars resistant to CBC[9]. Furthermore, quantitative results are available for diagnostic purposes with more practicable and unambiguous cutoff; for example, to determine *Xcc* positive or negative based upon the quantitative limit of detection and the rate of false positives.

The quantification of nucleic acids by qPCR method is an indirect method, which has several limitations; the basis of the methodology is to estimate the initial concentration of target DNA by comparing the value of the quantification cycle (*C*_q_) of a test sample to an external DNA calibrator[10]. The latter, in turn, is typically obtained from measuring a series of known standards (the positive plasmid DNA-carrying target sequence to be amplified) across the linear range of the assay. However, considerable variation in assay performance characteristics and in materials used as calibrators may prevent agreement between different laboratories, even when testing identical material[11].

Digital PCR is a novel molecular method enabling absolute quantification of DNA targets without the need to construct a calibration curve as used commonly in qPCR[12]. The principle of digital PCR was first introduced in the 1990s[12,13] and the recent development of droplet digital PCR (ddPCR) has been used widely in medical researches and clinical applications. The ddPCR approach partitions a bulk fluorescent PCR reaction containing DNA templates, primers and a fluorescently labeled hydrolysis probe or a nucleic acid intercalating dye (EvaGreen) into thousands of nanoliter-sized water-in-oil microdroplets. Target DNA and background DNA are distributed randomly among these droplets. Every microdroplet is a micro PCR reactor, with each containing zero or at least one copy of the target DNA[14–16]. After emulsion PCR to the end-point, droplets are analyzed individually by a mechanism similar to flow cytometry. Fluorescent and non-fluorescent droplets are defined as positive (presence of target sequence) or negative (absence of target sequence), respectively. The number of target DNA molecules present in the sample can be calculated from the fraction of positive droplets and Poisson statistics[14] using the following formula:

λ = –ln (1–p)

where *p* is the fraction of positive droplets and *λ* is copies per droplet. The concentration of target DNA can be calculated as:

Concentration = λ / V

where *V* is the average volume of a droplet. It is important to note no comparative external quantitative standard is needed to quantify unknown samples.

As an emerging versatile molecular biotechnology, ddPCR is a robust and powerful method for the detection and quantification of nucleic acids with unparalleled accuracy and precision without the need for an external calibration curve or reference. ddPCR is rapidly replacing qPCR as an efficient method for independent DNA quantification[17]. In recent years, there have been increasing numbers of applications[18] of the ddPCR assay used in medical, environmental[19–21] and food safety control applications[22–25], but a few related to plant pathogen diagnosis and phytopathology. In this work, we transferred an established and standardized qPCR assay for CBC detection directly to ddPCR format. We compared linearity, dynamic range, sensitivity, reproducibility, tolerance to inhibitors and diagnostic performance of both methods to assess the potential of ddPCR in diagnosis for CBC, aiming to provide a powerful new tool with higher accuracy and precision for detection of plant pathogens as well as phytopathology research.

## Materials and Methods

### Bacterial strain, media and growth conditions

The *Xanthomonas citri* subsp. *citri* culture used in this work belongs to the *Xcc* strain A isolated from infected citrus trees in Guangxi province, China. Bacteria were activated by cultivation at 28°C for 24 h on Luria-Bertani (LB) medium (pH 7.0) then at 28°C in liquid LB medium overnight to form a bacterial suspension. The number of colony-forming units (CFUs) of bacterial suspension was determined by plating on LB medium and counting the number of growing colonies. Briefly, LB medium was poured into 3 standard 90 mm plates. An overnight culture of *Xcc* was diluted and 100 μL were plated. The plated bacteria were grown overnight at 28°C and the number of growing colonies was counted manually. Then the *Xcc* bacterial suspension was serially diluted to generate a series consisting of nine tenfold dilution steps, which was used to test the analytical sensitivity, linearity and dynamic range of ddPCR and qPCR assays.

### Preparation of cloned plasmid standard

A DNA segment encoding the hypothetical conserved protein sequence of *Xcc* was amplified using the primer described below with *Xcc* genomic DNA as the template. The PCR amplicon was purified using the AxyPrep DNA Gel Extraction Kit (Axygen, USA), and ligated into the pMD 19-T vector system (TaKaRa, Japan). After transformation of the ligation products into *Escherichia coli* trans 5α chemically competent cells, positive colonies were identified among cultured single colonies grown at 37°C overnight in LB medium. An AxyPrep^™^ 96 Plasmid Kit (Axygen,) was used to extract plasmid DNA from a positive colony cultured in LB liquid medium at 37°C for 12–16 h. The concentration of plasmid DNA was measured using a spectrometer (Beckman, USA) and recalculated to obtain a value for plasmid copies/μL. The cloned plasmid DNA was linearized by endogenous restriction enzymes Hind III (FastDigest; ThermoFisher, USA) which does not target any sequence in the PCR amplicon. Enzymatic digestion of mixture comprised of 2 μL of FastDigest buffer (10×; ThermoFisher), 1 μL of Hind III restriction enzyme (ThermoFisher), 3 μL of plasmid DNA, and 14 μL of double-distilled water. The enzymatic reaction lasted for 1 h at 37°C and inactivated for 10 min at 80°C. The linearized plasmid DNA was used to generate a standard curve for serial dilutions consisting of nine tenfold dilution steps, which was also used in assessment of analytical sensitivity, linearity and dynamic range of ddPCR and qPCR assays.

### Sample collection

CBC field samples were collected from infected (confirmed previously) citrus trees in Guangxi province, China. Five sampling sites were located in north, west, south, east and central region of the orchard respectively and five infected trees were sampled at each site. Symptomatic and asymptomatic leaves were collected simultaneously from each infected citrus tree (total 50 infected samples, including 25 symptomatic and 25 asymptomatic samples). 32 *Xcc* negative leave were obtained from healthy citrus trees growing in our laboratories’ greenhouse. Individual samples were placed into a plastic bag, sealed to prevent cross contamination and stored at 4°C.

### Sample preparation

Citrus canker is mainly a leaf-spotting and rind-blemishing disease. Simple soaking and washing can isolate *Xcc* cells from the host citrus tissue. According to the instruction of the Chinese National standard for CBC diagnosis[26], five leaves of each sample were soaked in 10 mL of sterile PBS at room temperature with shaking at 150 rpm for 1 h and then centrifuged at 10,000 ×g for 10 min. The supernatant was discarded and 100 μL of the precipitate was stored at 4°C.

### Primers and fluorescently labeled hydrolysis probe

Primers and a fluorescently labeled hydrolysis probe were extracted from the Chinese National Standard for CBC diagnosis as follows:

sense primer; nucleotides 2617782–2617799, 5′-GGCTATTGGCTGACTTCA-3′

anti-sense primer; nucleotides 2717882–2617865, 5′-GATCCGTCCTCCATAACG-3′

probe; FAM-5′-2617862CTCGCAAGGCACGAATGCAA2617843-3′-BHQ1

The primers and probe were designed according to the *Xcc* conserved region encoding a hypothetical protein (GenBank CP011827.1; amplicon length 101 bp). The analytical specificity of the primers and the hydrolysis probe were verified during development and establishment of the quoted national standard of qPCR assay for CBC diagnosis. The primers and the probe were synthesized by Sangon Biotech (Shanghai), Co., Ltd. (China).

### Quantitative PCR assay

qPCR assays in the CFX Connect^™^ Real-time PCR System (Bio-Rad, Hercules, CA, USA) used optimized 20 μL reaction mixtures containing 2.5 μL of PCR buffer (10×; Takara), 1.5 μL of MgCl2 (25 mM; TaKaRa), 0.5 μL of dNTPs (10 mM each; TaKaRa), 0.5 μL of Taq DNA polymerase (5 U/μL; Promega), 1 μL of each primer, 1 μL of probe, 2 μL of *Xcc* sample and 10 μL of double-distilled water. The thermocycling protocol was: initial denaturation at 95°C for 5 min, then 40 cycles of denaturation at 95°C for 10 s, annealing at 60°C for 30 s. Standard curves constructed for serial dilutions of plasmid DNA were included in every qPCR run to produce quantitative results instead of raw Cq values. A no template control and standard curve were included in all runs and every sample was measured in triplicate.

### Droplet Digital PCR assay

The QX100^™^ Droplet Digital^™^ PCR System (Bio-Rad, Hercules, CA, USA) was used in this study according to the manufacturer’s instructions. Briefly, the ddPCR reaction mixtures (20 μL) contained: 1× ddPCR Supermix (Bio-Rad, Pleasanton, CA, USA), 750 nM of each primer, 750 nM of probe, 2 μL of *Xcc* sample. Droplets were generated using a Droplet Generator (DG) with an 8-channel DG8 cartridge and cartridge holder with 70 μL of DG oil/well, 20 μL of fluorescent PCR reaction mixture and a DG8 gasket. The prepared droplets were transferred to corresponding wells of a 96-well PCR plate (Eppendorf, Germany) using a multichannel pipette (Rainin, USA) by aspirating 40 μL from the DG8 cartridge. The PCR plate was subsequently heat-sealed with pierceable foil using a PX1^™^ PCR plate sealer (Bio-Rad) and then amplified in a C1000 Touch^™^ deep-well thermal cycler (Bio-Rad). The thermocycling protocol was: initial denaturation at 95°C for 5 min, then 45 cycles of denaturation at 95°C for 30 s, annealing at 54°C for 45 s (temperature ramp 2°C/s) and, finally, incubation at 98°C for 10 min and storage at 4°C. After cycling, the 96-well plate was fixed into a plate holder and placed into the Droplet Reader (Bio-Rad). Droplets of each sample were analyzed sequentially and fluorescent signals of each droplet were measured individually by a detector.

### Thermal gradient optimization of ddPCR assay

To assess the optimal annealing temperature of the ddPCR assay, the temperature gradient was performed in the C1000 Touch^™^ deep-well thermal cycler by replacing the annealing temperature step of the standard ddPCR cycling program with a thermal gradient between 53°C and 68°C for 45 s extension time. Eight ddPCR reactions containing the same amount of *Xcc* DNA were annealed at different annealing temperatures.

### Assessing inter-assay variability between ddPCR and qPCR assays for independent experiments

To evaluate and compare the reproducibility of the qPCR and ddPCR assays, triplicate experiments for both ddPCR and qPCR assays were performed as independent replicates from two bacterial suspension samples and six plasmid DNA samples. The samples were assayed in three different runs of both platforms. The coefficient of variation (CV) for each sample with triplicate qPCR and ddPCR assays was calculated to reflect the reproducibility between runs.

### Estimation of tolerance to inhibitors

The tolerance of qPCR and ddPCR to common inhibitors in citrus samples was estimated with reactions containing different concentrations of inhibitors. These reactions were spiked with the same amount of *Xcc* DNA and the resilience was calculated as the ratio of the target concentration in the presence of different amounts of inhibitors to the absence of inhibitors (no-inhibition control, as double-distilled water plus *Xcc* DNA). Citrus extracts and CuSO4 (Sigma-Aldrich, USA) as well as cupravits (copper-containing bactericide) were used to test tolerance of the ddPCR and qPCR assays. To obtain citrus extracts, healthy citrus leaves were sheared into pieces, soaked in TE buffer (pH 7.4) and incubated at room temperature overnight. The mixture was centrifuged at 10,000 ×g for 5 min and the green supernatant was recovered to test the tolerance of both the qPCR and ddPCR assays to the citrus extracts.

### Data analysis

The qPCR Cq values and standard curves were generated by CFX^™^ Manager Software version 3.1 (Bio-Rad). Linear regression of the qPCR standard curves was recalculated with Microsoft Excel software (Microsoft, USA). For ddPCR, fluorescent signals of droplets were manipulated with the QuantaSoft^™^ version 1.7 (Bio-Rad). Positive droplets with higher fluorescent signals and negative droplets with lower fluorescent signals were divided by applying a fluorescence amplitude threshold between them. The copy number concentration of each sample was reported automatically by ddPCR software. The linear range of the ddPCR assay was determined by plotting the measurements of ddPCR and comparing them with expected values of serial dilution of plasmid DNA and bacterial suspension. Pearson’s correlations and linear regression were also used to evaluate the relationship between measurements of ddPCR and qPCR assays. To compare the differences in measurements of ddPCR and qPCR assays between the healthy and infected groups, t-tests were used. Receiver operating characteristic (ROC) curves were constructed to evaluate the diagnostic performance of the ddPCR and qPCR assays. Statistical analyses were performed with GraphPad Prism 6 software (GraphPad Software, Inc., San Diego, CA, USA).

### Ethics statement

Field CBC samples were collected from infected citrus trees (confirmed previously) growing in the Hepu Plantation in the Guangxi province of China. The plantation (N21°43'3.2988" E108°59'40.9704") belongs to Lucky Team Biotech Development (Hepu) Co., Ltd. (Tel (86)-779-7198851; Fax (86)-779-7122059). The authors were granted permission to collect the diseased leaf samples. There was no protected species growing within the sample area.

## Results

### Optimization of the ddPCR assay

**Fig 1:**
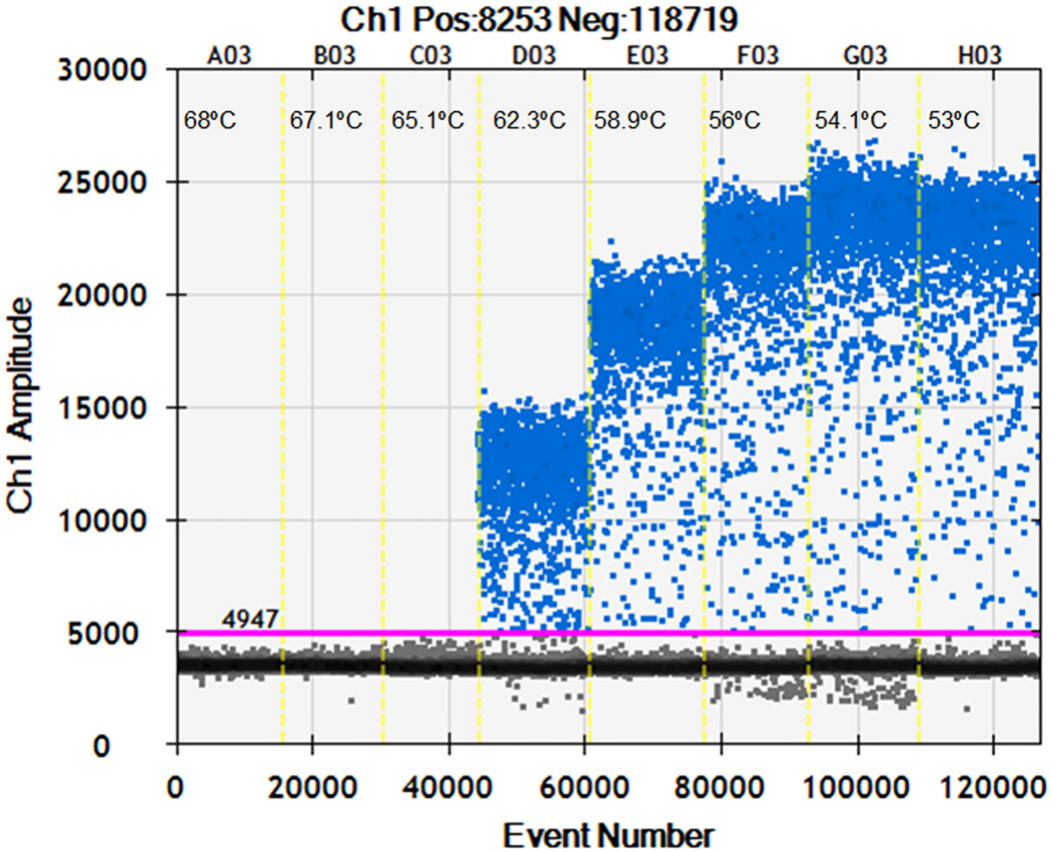
Fluorescence amplitude plotted against annealing temperature gradient. The unbroken pink line is the threshold, above which are positive droplets (blue) with PCR amplification and below which are negative droplets (gray) without any amplification. Eight ddPCR reactions with the same amount of targets are divided by the vertical dotted yellow line. The reactions were across an annealing temperature gradient: 53, 54.1, 56, 58.9, 62.3, 65.1, 67.1 and 68.0°C. The optimal range of annealing temperatures giving the largest difference in fluorescence between negative and positive droplets was between 52°C and 55.4°C.

To determine the optimum annealing temperature for the ddPCR assay, temperature gradient was set at the following eight temperatures: 53, 54.1, 56, 58.9, 62.3, 65.1, 67.1 and 68.0°C on the thermal cycler. The optimal range of annealing temperatures giving the largest difference in fluorescence between negative and positive droplets was between 53°C and 56°C. An optimized annealing temperature of 54°C was chosen for the subsequent ddPCR tests (Fig 1).

### Comparison of analytical sensitivity, linearity and dynamic range between ddPCR and qPCR assays

**Fig 2:**
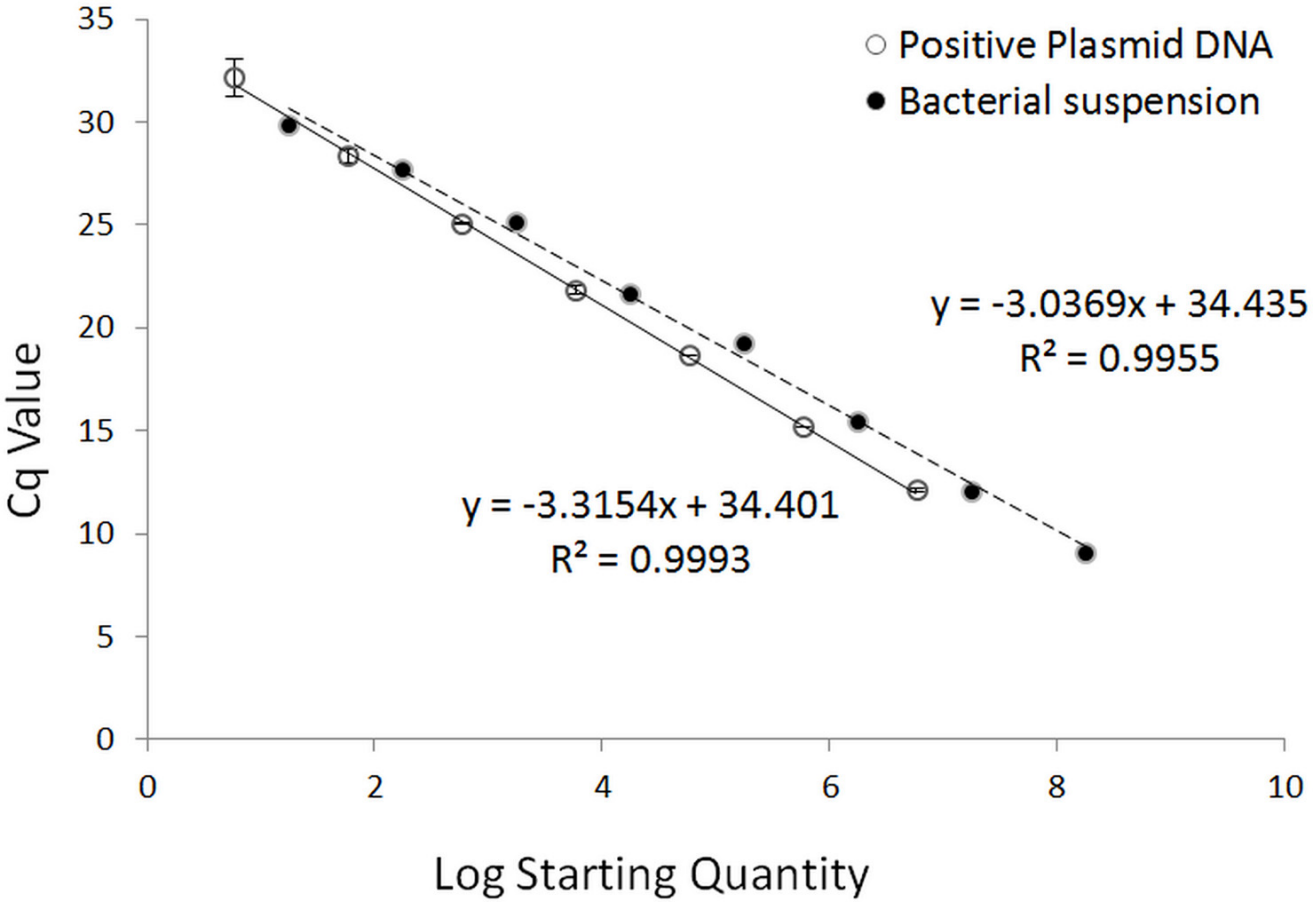
Calibration curves of qPCR assays run with positive plasmid DNA (unbroken line) and bacterial suspension (broken line). Plasmid DNA was tenfold diluted serially from 5.88E+6–5.88E+0 copies/μL. The slope of the plasmid DNA standard curve is –3.3154, equivalent to an efficiency of 100.3% (*R*^2^ = 0.9993). The bacterial suspension was 10-fold serially diluted from 1.78E+8–1.78E+1 CFU/μL. The slope of the bacterial suspension calibration curve is –3.0369, equivalent to an efficiency of 113.5% (*R*^2^ = 0.9955), indicating PCR inhibition probably caused by the residual medium matrix.

**Fig 3:**
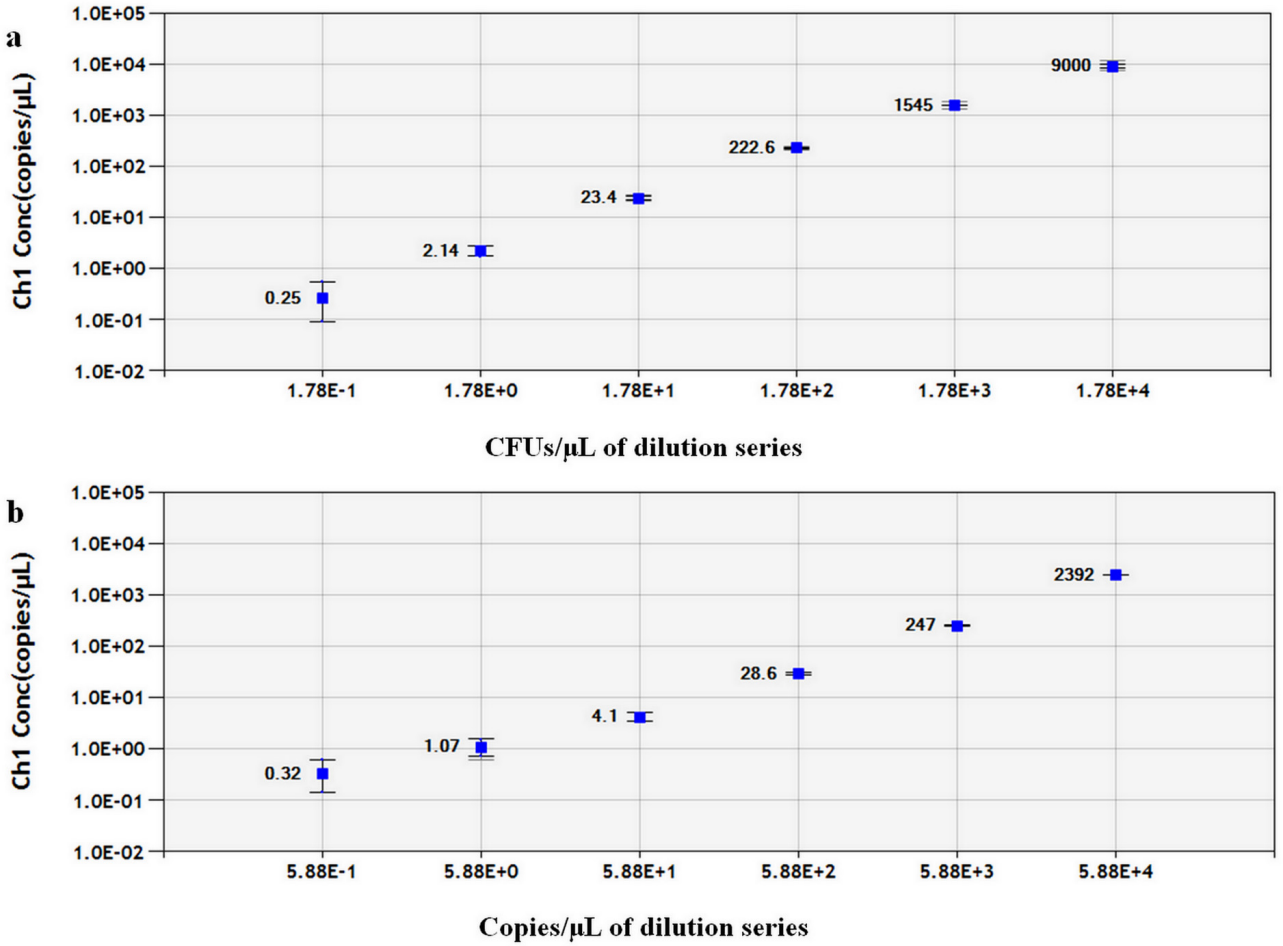
Linear regression of the ddPCR assay for (a) bacterial suspension and(b) positive plasmid DNA constructed by the same serial dilution series tested with the qPCR assay (see Fig 2). The estimated Pearson correlation coefficient of the bacterial suspension regression curve (y = 1.9902x—283.81) is 0.995 (*R*^2^ = 0.995, *P*< 0.0001) and that of the plasmid DNA regression curve (y = 24.607x—74.083) is 1.0 (*R*^2^ = 1.0, *P*< 0.0001). Both standards tested by ddPCR exhibited a dynamic range of five orders of magnitude. The vertical axis shows the log_10_-transformed copy number/μL of the ddPCR reaction mixture. The horizontal ordinate indicates (a) the log_10_-transformed expected concentration of CFU/μL of the ddPCR reaction mixture or (b) the log_10_-transformed expected copy number/μL of the ddPCR reaction mixture. The inner error bars indicate the Poisson 95% confidence interval (CI) and the outer error bars show the total 95% CI of replicates.

Tenfold serial dilution series of both positive plasmid DNA and *Xcc* bacterial suspension were used to construct the calibration curves for the qPCR assay (Fig 2) and the regression curves for the ddPCR assay (Fig 3) for comparison of the analytical sensitivity, linearity and dynamic range of the two assays. The qPCR assay exhibited good linearity (R2 = 0.999 and 0.9955 for plasmid DNA and bacterial suspension series, respectively) over the dynamic range tested in both the positive plasmid DNA (5.88E+6–5.88E+0 copies/μL) and the bacterial suspension standards (1.78E+8–1.78E+1 CFU/μL). In the qPCR standard curves, the slopes were –3.3154 for the positive plasmid DNA and –3.0369 for the bacterial suspension, equivalent to a PCR efficiency of 100.3% and 113.5%, respectively. It is worth noting the efficiency of the plasmid DNA qPCR test was essentially perfect (100.3%), whereas the efficiency of the bacterial suspension qPCR test was greater than 100% (113.5%), indicating PCR inhibition, which was probably caused by the residual LB liquid medium in the bacterial suspension dilutions. According to the standard curves, the sensitivity of the qPCR test for plasmid DNA and bacteria cells was approximately 12 copies/20 μL reaction and 36 CFUs/20 μL reaction, respectively. The Cq values and logarithmic starting concentrations of qPCR serial dilution series are shown in S1 Table.

**Fig 4:**
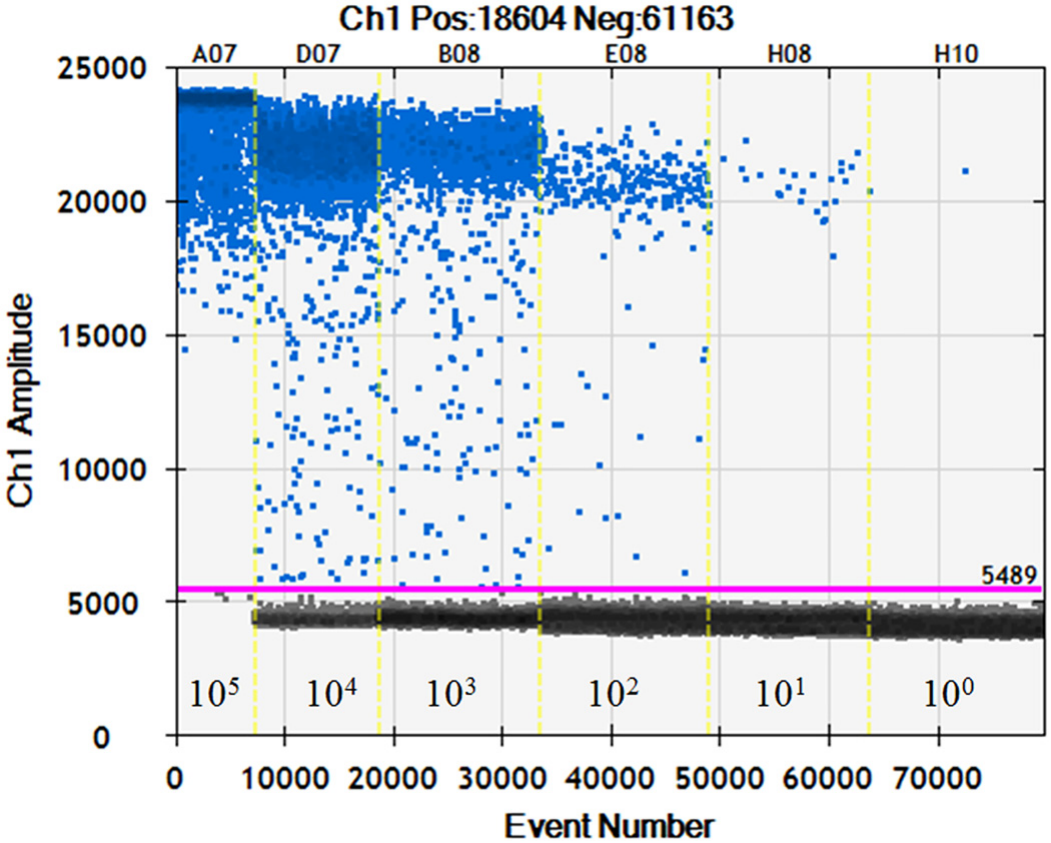
Representative 1-D plot of ddPCR reactions. The ordinate scales indicate fluorescent amplitude. The unbroken pink line is the threshold, above which are positive droplets (blue) containing at least one copy of target DNA and below which are negative droplets (gray) without any target DNA. Six ddPCR reactions with various serially diluted targets are divided by the vertical dotted yellow line. The leftmost ddPCR reaction was saturated by an excess target concentration and the rightmost reaction contains a single copy of the target.

Quantitative linearity of ddPCR assay was also assessed by quantification of bacterial suspension and plasmid DNA standards. The log10-transformed copy numbers concentration measured by ddPCR were plotted against the corresponding log10-transformed predicted values of serially diluted bacterial suspension and plasmid DNA and fitted with a linear regression mode. The measurements of ddPCR assay showed both bacterial suspension (Fig 3a) and positive plasmid DNA standards (Fig 3b) exhibited good linearity (R2 = 0.995 and 1 respectively, P<0.0001) between the target input amounts and measured values in a dynamic range of five orders of magnitude. Droplets were positively saturated at target concentrations ≥10^6^ copies/μL, making the Poisson algorithm invalid and resulting in a relative narrower dynamic range compared to qPCR. In this study, the sensitivity of the ddPCR assay for plasmid DNA was down to 6.4 copies/20 μL reaction, which is more sensitive than qPCR. The ddPCR assay for direct detection of bacterial suspension showed better sensitivity compared to the qPCR assay, because positive droplets could be obtained in 5 CFUs/20 μL reactions. A representative one dimensional (1-D) plot of ddPCR reactions with serially diluted targets is shown in Fig 4. Copy numbers of serial dilution series detected by the ddPCR assay are given in S2 Table.

### Assessment of reproducibility of the qPCR and ddPCR assays

**Fig 5:**
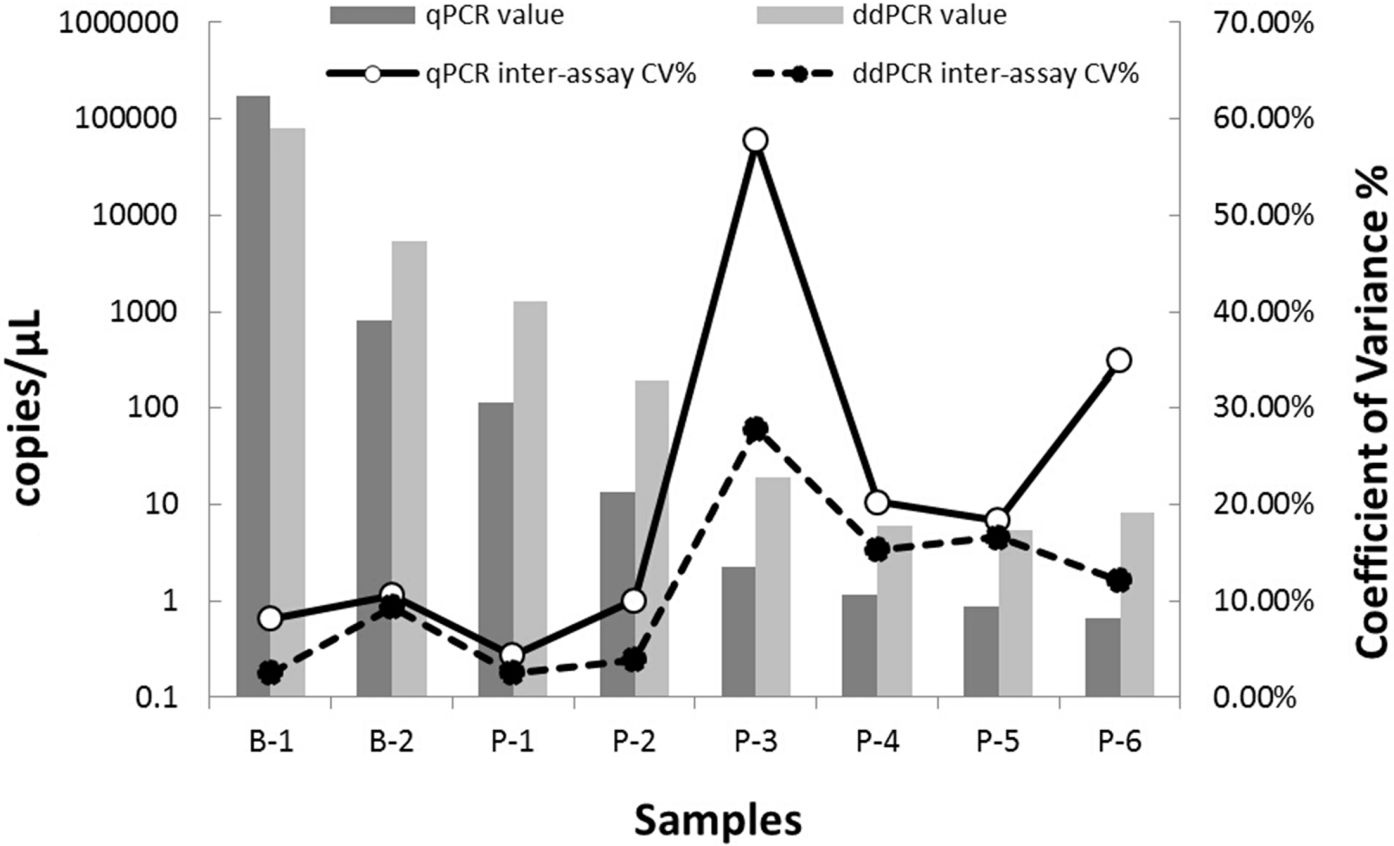
Inter-assay CV% of the qPCR and ddPCR assays. Samples B-1 and B-2 are bacterial suspensions in high concentration. Samples P-1 –P-6 are positive plasmid DNA; among them, P-1 and P-2 are of high concentration and P-3 –P-6 are of low concentration. Histograms indicate the average copy number of each sample in log 10 scale. Lines show the trend of variation of CV of the qPCR and ddPCR assays with repeated tests of diverse sample concentrations. The ddPCR assay is more precise compared to the qPCR assay for quantification of *Xcc*, especially for low target concentrations (numerical data supporting Fig 5 are given in S3 Table).

As shown in Fig 5, comparison of *Xcc* quantification by the ddPCR and qPCR assays revealed improved reproducibility between runs by ddPCR (CV% decreased by 9.19%–69.44%; numerical data supporting Fig 5 are given in S3 Table), especially for samples of low target concentrations (<10 copies/μL).

### Influence of inhibitors on the qPCR and ddPCR assays

**Fig 6:**
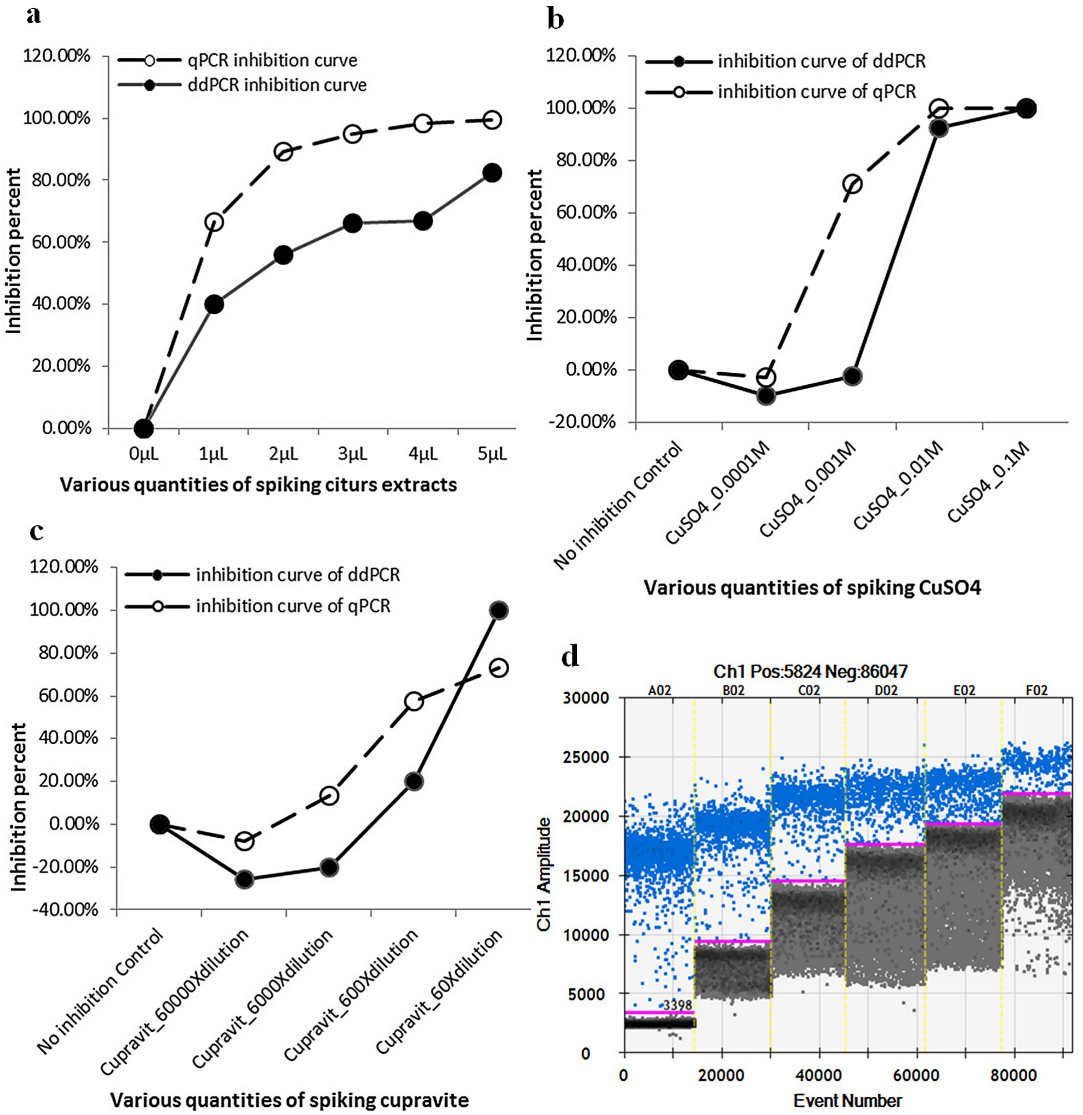
Influence of samples spiked with serial dilutions of an inhibitor on quantification by the qPCR and ddPCR assays. The ddPCR assay exhibits superior tolerance to citrus extracts and Cu^2+^ compared to the qPCR assay. A 100% inhibition represents a completely suppressed reaction with no positive signal and a 0% inhibition indicates no suppression with the same target concentration as the no-inhibition control. (a) Citrus extract, (b) CuSO_4_ and (d) cupravit had an enhancing effect on both the ddPCR and qPCR assays at low levels of spiking. (d) A 1-D plot of ddPCR reactions spiked with different amounts of citrus extracts. Fluorescent signals of both positive and negative droplets were increased with increasing amounts of citrus extracts.

The resilience of qPCR and ddPCR assays to citrus extracts and copper component was estimated with reactions containing different concentrations of inhibitors. These reactions were spiked with the same amount of *Xcc* DNA and the resilience was calculated as the ratio of the target concentration in the presence of different amounts of inhibitors to the absence of inhibitors (Fig 6). In citrus extracts and CuSO_4_ tests, the qPCR suppression curve was above that of ddPCR, indicating the qPCR assay was more susceptible to the two inhibitors (Fig 6a and 6b). In a test of cupravit, the suppression curve of ddPCR was beneath that of qPCR but reached 100% inhibition before qPCR at 60-fold diluted cupravit (Fig 6c). This was because droplet generation was disabled because of a channel in the droplet generation cartridge being blocked by the high concentration of cupravit. Although high concentrations of copper inhibited ddPCR and qPCR, it appeared copper (Cu^2+^) has a positive effect on both ddPCR and qPCR assay with low level of spiking (Fig 6b and 6c). The ddPCR assay combined with increasing amounts of citrus extracts exhibited increased fluorescent signals for both negative and positive droplets (Fig 6d), which might be owing to increased concentrations of chlorophyll from citrus leaf tissues.

### Comparison of diagnostic performance between the ddPCR and qPCR assays

**Fig 7:**
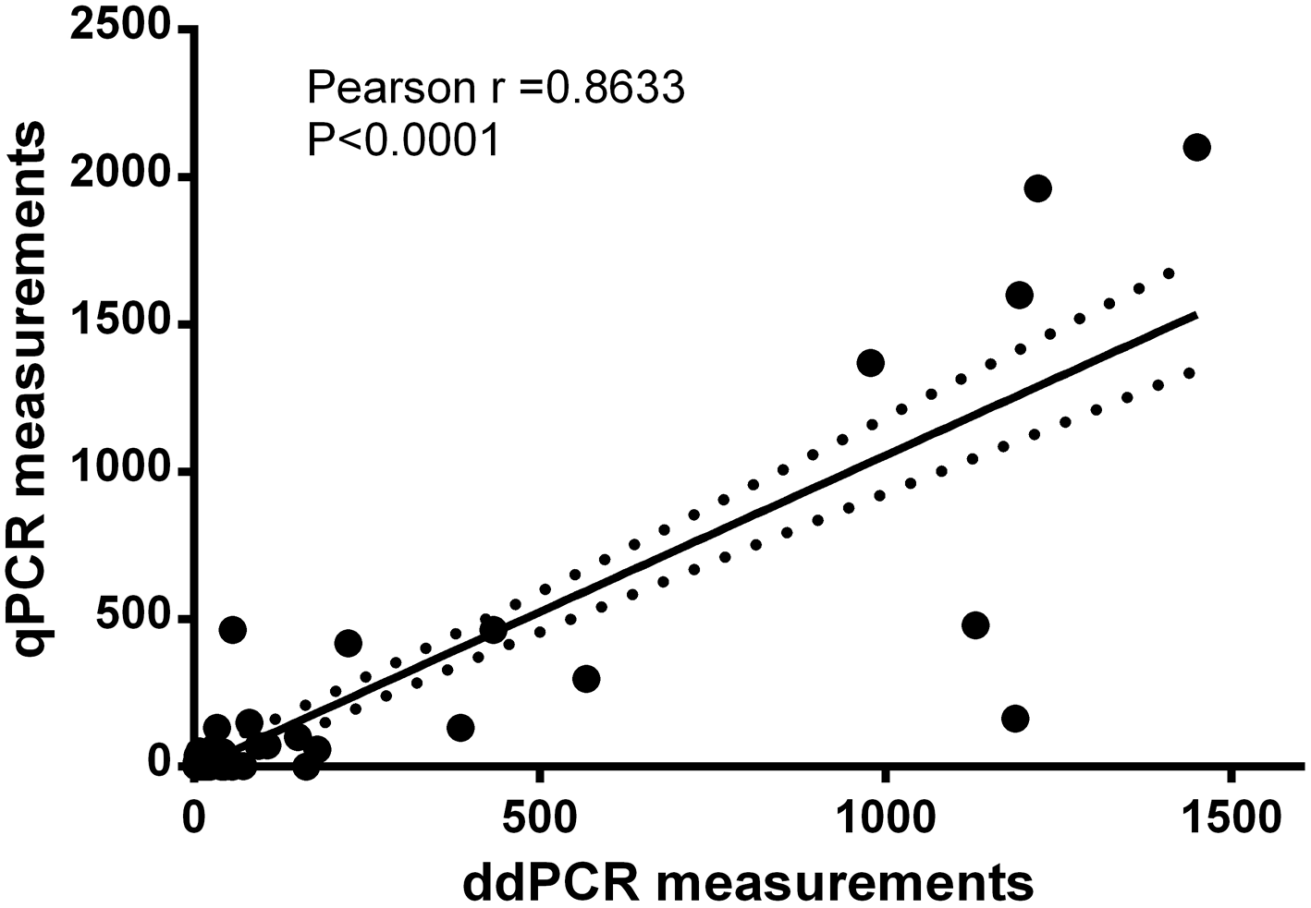
Correction of ddPCR and qPCR measurements. Measurements of citrus samples by ddPCR and qPCR assays were significantly correlated (Pearson r = 0.8633, P<0.0001). Solid line indicates fitting curve; dashed line represents 95% CI.

Fifty citrus samples from infected area and thirty-two citrus samples from healthy trees were tested by ddPCR and qPCR assays to determine whether ddPCR assay can replace the qPCR assay. The linear association for *Xcc* measurements between ddPCR and qPCR assays is strong based on Pearson's correlation (*R*^*2*^ = 0.7453; *P* <0.001). Moreover, the slope and intercept of *Xcc* by ddPCR to qPCR assay in the linear regression were significant (*P* <0.0001) with a value equal to 1.062 (standard errors of estimates = 0.069) and -5.770 (standard errors of estimates = 23.63), respectively (Fig 7).

*Xcc* was detected in 46/50(92%) infected citrus samples, including 25/25(100%) symptomatic samples and 21/25(84%) asymptomatic samples. 31/50(62%) infected citrus samples were positive by qPCR, including 22/25(88%) symptomatic samples and 9/25(36%) asymptomatic samples (Table 1). Of note, the *Xcc* detected in the citrus samples by ddPCR were not completely matched with those by qPCR (Table 2), the concordance between the ddPCR assay and qPCR assay was 34 of 50 samples (70%). The ddPCR assay showed higher positive predictive value compared to the qPCR assay, indicating that ddPCR is more robust method for detection of *Xcc* in asymptomatic citrus tissue in latent period of infection.

**Table 1:** Performance of ddPCR and qPCR assay for detection of *Xcc* in symptomatic and asymptomatic infected citrus samples.

Infected citrus sample	ddPCR		qPCR	
	Positive	Negative	Positive	Negative
Symptomatic	25	0	22	3
Asymptomatic	21	4	9	16

**Table 2:** Correlation of infected citrus samples between ddPCR and qPCR assay.

Infected citrus sample by qPCR	Infected citrus sample by ddPCR	
	Positive	Negative
Positive	31	0
Negative	15	4

**Fig 8:**
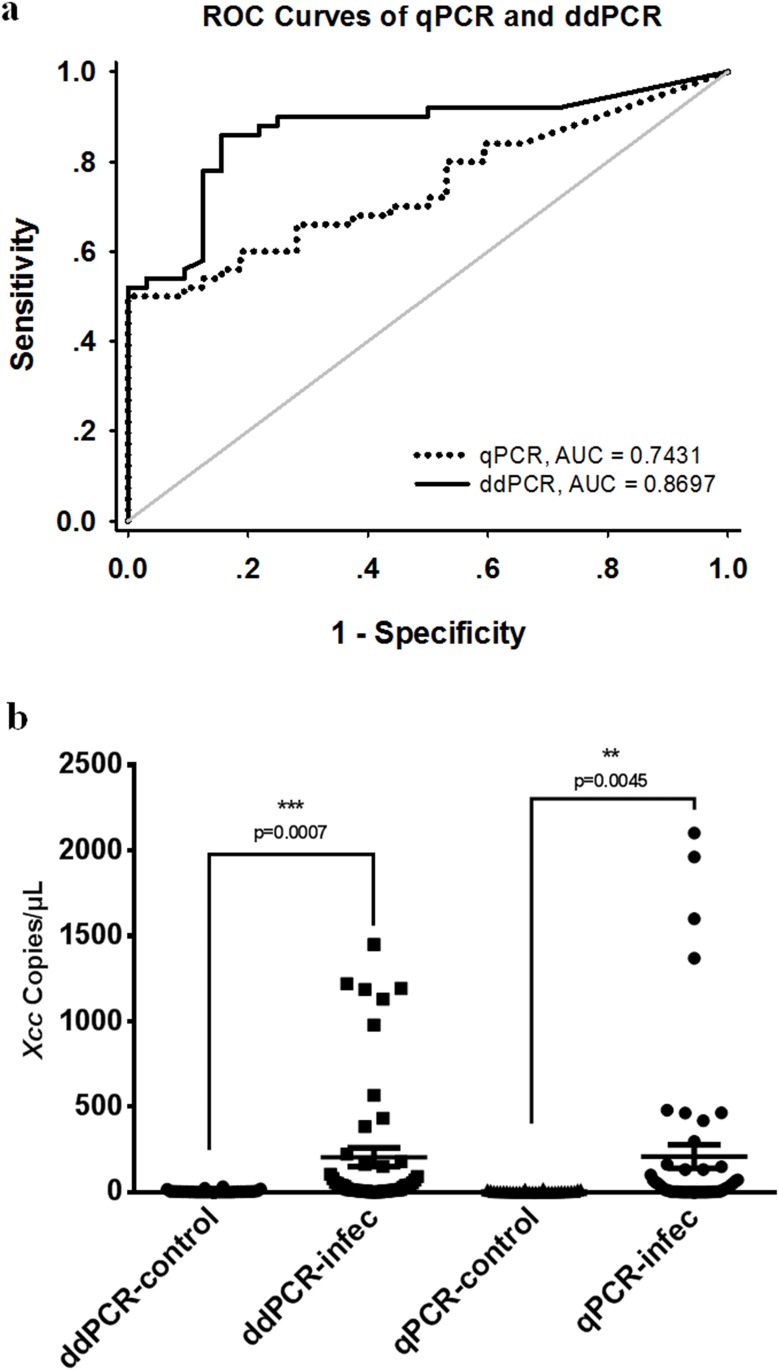
Diagnostic performance of *Xcc* quantification by the ddPCR and qPCR assays. ROC and AUC were used to estimate the sensitivity and specificity of each method. AUC for each assay indicated its performance in differentiating CBC-infected trees from the control cohort in terms of sensitivity and specificity. (a) ROC curves of ddPCR and qPCR assays. The ddPCR assay indicates better diagnostic performance compared to the qPCR assay. (b) T-test shows both ddPCR and qPCR assays can discriminate significantly between healthy control and *Xcc*-infected citrus samples.

ROC curves for the ddPCR and qPCR assays were constructed with the quantitative results of citrus samples including healthy citrus leaves and symptomatic and asymptomatic leaves on infected citrus trees in the field, respectively. The value of AUC for each assay indicated its performance in differentiating CBC-infected trees from the control cohort in terms of sensitivity and specificity. As shown in Fig 8a, the ddPCR assay had an AUC of 0.8697 (*P* <0.0001, standard error 0.04074, 95% CI 0.7898–0.9495) and the qPCR assay had an AUC of 0.7431 (*P* = 0.0002, standard error 0.05288, 95% CI 0.6395–0.8468). Although both qPCR and ddPCR assays can discriminate significantly between healthy control and infected citrus samples (Fig 8b), the AUC of the ddPCR assay is significantly (*p* <0.05) broader compared to the qPCR assay, indicating the ddPCR methodology is a more robust approach for CBC diagnosis compared to qPCR.

## Discussion

*Xcc*, the causal agent of canker in citrus, is a well-known and serious pathogen worldwide causing economically important diseases of citrus industry. Advances in diagnosis of CBC have been made through the application of qPCR technology, which offers sensitive, reliable, and fast detection and identification of *Xcc* as well as other plant pathogens, however quantitative determinations by qPCR are indirect, depending on the relationship of the Cq of a test sample to a calibration curve. Cq varies according to different threshold positions as well as PCR efficiency; a sample can have various Cq values corresponding to variable threshold positions within the exponential phase even in the same run. Therefore, even qualitative determination based on Cq is either subjective or empirical, and it is less rigorous to draw a cutoff line simply using the raw Cq value. The external calibration curve for absolute quantification is usually obtained from a series of dilutions of known concentrations, which is either expensive because metrological standard reference materials are needed, or laborious and time-consuming to put onto place[27]. In addition, the reliability and consistency of the standards greatly affect the accuracy of quantification of the unknown samples by the qPCR assay. For example, the degradation of reference DNA materials that can occur during storage usually results in overestimation of unknown samples[28]. Furthermore external DNA calibrators from various sources and differences between batches as well as storage and handling conditions can make quantitative results vary from day to day within a laboratory and between laboratories. Moreover, the qPCR assay is sensitive to PCR inhibitors, which can decrease the efficiency of PCR and thus cause shifts of Cq toward larger values leading to underestimation of concentrations. The presence of inhibitors has the potential to increase error, reduce assay resolution and produce misleading results, including false negative results in both quantitative and qualitative PCR assays[29].

The recently developed ddPCR technology offers several advantages that conventional qPCR lacks, including (i) improved sensitivity[30,31], (ii) absolute quantification without the need for an external calibration curve, (iii) less susceptible to PCR inhibitors[32,33] and variation of PCR efficiency, (iv) increased precision, especially in low concentration samples[30,34,35], (v) improved accuracy, reliability and between run reproducibility, and (vi) more powerful discrimination between small changes of concentration[36].

In this study, cloned plasmid standard, *Xcc* bacterial suspension and citrus samples were quantified with ddPCR and qPCR assays. We compared the linearity, sensitivity, reproducibility and tolerance to PCR inhibitors as well as diagnostic performance of ddPCR assay with qPCR assay, to evaluate the feasibility of using the novel method of ddPCR to quantitative detection of *Xcc*. The correlation between ddPCR measurements and predicted values was good for both plasmid and bacterial suspension (Fig 3a and 3b). However, it is worth noting the ddPCR measurements of each dilution differed from the expected value of either plasmid DNA or bacterial suspension. The ddPCR assay determined fewer target sequence copies than expected by the calculated plasmid DNA copy number but more target copies compared to the corresponding CFU value (S2 Table). This result was owing to a methodological discrepancy between the ddPCR and spectrophotometer method and the CFU method. The expected copy number of positive plasmid DNA was measured by ultraviolet detection and then recalculated by an empirical formula; however, this method lacks accuracy because measurement of optical density cannot distinguish between intact and fragmented nucleic acids. Additionally, the CFU method relies on the ability of bacterial cells to form colonies. Dead cells cause reduced CFU counts and both dead bacterial cells and living cells can be amplified in PCR and defined as positive. It is worth noting the ddPCR assay provides reliably accurate quantification of nucleic acids and is accepted for the preparation of metrological standard DNA reference materials[37–41]. In ddPCR amplification using intact bacterial cells directly, bacteria are encapsulated into droplets as intact units. Subsequently, DNA was released into the aqueous phase within droplets at the initial denaturation stage at 95°C for 5 min. Therefore, the copy number polymorphism (also termed copy number variation) of the amplicon sequence among the *Xcc* population did not cause overestimation of the bacterial cell number in the ddPCR assay; however, this does occur with the qPCR assay, because multiple copies of target in one genomic of a bacterial cell released into bulk reaction of qPCR assay, increasing the total original amount of amplifiable target sequence[21].

In comparison of reproducibility of the two methods, without the need for an external standard curve, the ddPCR assay exhibited superior reliable and repeatable quantitative results compared to the qPCR assay, especially under conditions of low target concentration. The Cq value for a sample with a low target concentration is typically greater than 30, above which it is difficult to restrict standard deviation of technical replicates of the qPCR assay within 0.25 cycle, which is required to discriminate a twofold change of target concentration with 95% confidence interval (CI). The qPCR assay is an indirect method of extrapolating from the original concentration of the target from fluorescent signals to that directly proportional to the amount of PCR products. Factors with negative effects on PCR efficiency can make extrapolating from the fluorescent signals procedure of qPCR unstable. Additionally, single or few target molecules in a bulk qPCR reaction can lose PCR efficiency owing to the reduced probability of stochastically binding primers (and probe) together with polymerase in the target sequence of the template. The ddPCR method encapsulates a single or few target molecules into discrete nanoliter-sized micro reactors (droplets), however, enabling successful amplification with relatively higher target concentrations; imagine comparison of a few copies in a volume of 20 μL and the same number of copies in a nanoliter reaction. Taken together, our results showed the ddPCR approach is potentially more precise and reproducible compared to the qPCR approach. This is in accordance with existing reports[34–36].

Complex components, such as chlorogenic acid, in citrus tissue might be co-purified with bacterial DNA during sample preparation, which often inhibit PCR and lead to reduced sensitivity of the PCR assay used to detect *Xcc* in plant tissue, even giving false negative results. In addition, residual copper-containing bactericides such as cupravit, commonly used as an agricultural spray on citrus leaves, would also inhibit PCR[42]. Cupravit contains finely divided copper hydroxychloride (30% (w/v) suspension was used in this study), which is difficult to dissolve in water. High concentrations of undissolved copper hydroxychloride in the ddPCR assay can obstruct the microfluidic channels of a DG8^™^ cartridge, resulting in no droplet being generated. So we also tested copper sulfate (CuSO4) as a PCR inhibitor to assess the tolerance to the copper component. Unlike the qPCR assay, the ddPCR assay uses a digital mechanism for direct measurement of target nucleic acids. Owing to the virtue of end-point fluorescent signals collection and binomial events (positive or negative) counting associated with the Poisson algorithm, ddPCR is less susceptible to PCR inhibitors and variation of PCR efficiency. The decrease of PCR efficiency results in a Cq shift causing underestimation of target concentration, whereas in ddPCR, the reduction of amplifying efficiency results in a decrease of fluorescent signals of positive droplets. The ddPCR assay remains stable, however, in spite of the decrease of fluorescent signals with increasing amounts of PCR inhibitors until discrimination between positive and negative droplets becomes ambiguous. The ddPCR assay is more suitable for the detection of pathogens in field samples with a complex matrix of inhibitors. Even analysis of laboratory samples will benefit from the excellent tolerance of ddPCR to inhibitors and lower degree of susceptibility to variation of PCR efficiency.

ROC analysis, which is widely used in medical research and clinical applications, is a useful tool for evaluating the performance of a diagnostic approach. Samples are categorized into diseased and healthy groups and the analysis functions as a two-dimensional graph to provide the most comprehensive description of diagnostic specificity and sensitivity. For ROC, the area under the curve (AUC) is an intuitive value to exhibit an overall summary of diagnostic accuracy. AUC ranges from 0.5–1.0, where 1.0 represents complete accuracy. In this study, ROC analysis indicated the ddPCR assay is a more robust approach for CBC diagnosis compared to qPCR assay with better diagnostic performance. This might be owing to better sensitivity of ddPCR.

Overall, the development of ddPCR technology provides a more robust alternative method for quantitative detection of plant pathogen. Routine diagnosis and quarantine of plant pathogen may benefit from the direct quantification of ddPCR with more precise and reproducible results between las, eliminating the need for quantitative reference material, which is laborious and time-consuming to prepare. In other words, ddPCR assay also has the potential for accurate quantification of DNA copy number of certified reference materials for plant pathogen test. Findings in this study may also be of interest to other fields of research of plant pathogens, including epidemiological studies, analysis of bacterial infection kinetics, and determination of biologically relevant threshold.

## Conclusion

To the best of our knowledge, this work is the first to demonstrate how to apply the ddPCR technology to quantify *Xcc*. We transferred a routinely used qPCR assay for CBC diagnosis to the ddPCR format and evaluated the potential of the ddPCR assay compared to the established qPCR assay. The results demonstrated the ddPCR assay was a reliable alternative method for the quantitative detection of *Xcc*; however, this work is an example of ddPCR for detection of plant disease, further studies are required to evaluate and validate the value of the new technology for routine use in the quarantine and diagnosis of plant disease.

## Supporting Information

S1 Checklist MIQE checklist for both ddPCR and qPCR. (XLS)

S1 Table qPCR quantitative detection of plasmid DNA standard and bacterial suspension standard. (PDF)

S2 Table ddPCR quantitative detection of plasmid DNA standard and bacterial suspension standard. (PDF)

S3 Table Quantitative results of qPCR and ddPCR method in reproducibility comparison. (PDF)
