# Supplementary material for: Comparison of Droplet Digital PCR and Quantitative PCR Assays for Quantitative Detection of Xanthomonas citri Subsp. citri
Source: PLoS One. 2016 Jul 18;11(7):e0159004. doi: 10.1371/journal.pone.0159004 (PMC4948846; doi:10.1371/journal.pone.0159004)
Supplement: S1 Table — (PDF) [file pone.0159004.s002.pdf]

S1 Table. qPCR quantitative detection of plasmid DNA and bacterial suspension standards

Plasmid DNA[a]	Cq		Log SQ[b]	Bacterial suspension[c]	Cq		Log SQ
	Mean	SD			Mean	SD	
5.88E+6	12.12	0.032	6.769	1.78E+8	9.08	0.052	8.25
5.88E+5	15.16	0.001	5.769	1.78E+7	12.06	0.052	7.25
5.88E+4	18.68	0.006	4.769	1.78E+6	15.4	0.071	6.25
5.88E+3	21.83	0.097	3.769	1.78E+5	19.21	0.07	5.25
5.88E+2	25.06	0.005	2.769	1.78E+4	21.67	0.05	4.25
5.88E+1	28.34	0.186	1.769	1.78E+3	25.13	0.025	3.25
5.88E+0	32.15	0.46	0.769	1.78E+2	27.7	0.112	2.25
NTC[d]	0	0	0	1.78E+1	29.83	0.012	1.25
				1.78E+0	0	0	0
				NTC	0	0	0

[a] Values reflect copies/µL of calculated serial dilutions of positive plasmid DNA standard.

[b] SQ means starting quantity. Data represent the means of each dilution tested in triplicate.

[c] Values reflect CFUs/µL of calculated serial dilutions of *Xcc* bacterial suspension standard.

[d] NTC means no template control
