# Supplementary material for: Comparison of Droplet Digital PCR and Quantitative PCR Assays for Quantitative Detection of Xanthomonas citri Subsp. citri
Source: PLoS One. 2016 Jul 18;11(7):e0159004. doi: 10.1371/journal.pone.0159004 (PMC4948846; doi:10.1371/journal.pone.0159004)
Supplement: S2 Table — (PDF) [file pone.0159004.s003.pdf]

S2 Table. ddPCR quantitative detection of plasmid DNA and bacterial suspension standards

Plasmid DNA[a]	ddPCR results			Bacterial suspension[d]	ddPCR results		
	Mean[b]	Poisson SEM[c]	Total SEM		Mean[e]	Poisson SEM	Total SEM
5.88E+4	2392	18.88	18.88	1.78E+4	9000	459.18	1045.92
5.88E+3	247	3.57	3.57	1.78E+3	1545	9.18	132.65
5.88E+2	28.6	1.10	1.10	1.78E+2	222.6	2.55	6.89
5.88E+1	4.1	0.41	0.41	1.78E+1	23.4	0.81	1.71
5.88E+0	1.07	0.21	0.23	1.78E+0	2.14	0.24	0.24
5.88E-1	0.32	0.12	0.12	1.78E-1	0.25	0.11	0.11
NTC[f]	0	0	0	NTC	0	0	0

[a] Values reflect copies/μL of calculated serial dilutions of positive plasmid DNA standard.

[b] Values reflect copies/μL ddPCR reaction. Data represent the ddPCR measurement from merged triplicate tests of each dilution.

[c] SEM means standard error of mean.

[d] Values reflect CFUs/μL of calculated serial dilutions of *Xcc* bacterial suspension standard.

[e] Values reflect CFUs/μL ddPCR reaction. Data represent the ddPCR measurement from merged triplicate tests of each dilution.

[f] NTC means no template control.
