# Supplementary material for: Comparison of Droplet Digital PCR and Quantitative PCR Assays for Quantitative Detection of Xanthomonas citri Subsp. citri
Source: PLoS One. 2016 Jul 18;11(7):e0159004. doi: 10.1371/journal.pone.0159004 (PMC4948846; doi:10.1371/journal.pone.0159004)
Supplement: S3 Table — (PDF) [file pone.0159004.s004.pdf]

S3 Table. Quantitative results of qPCR and ddPCR method in reproducibility comparison

Sample ID	qPCR[a]				ddPCR[b]				CV% Decreased[c]
	Test 1	Test 2	Test 3	CV%	Test 1	Test 2	Test 3	CV%	
B-1	181957.12	155960.57	178315.12	8.18%	80000	82000	78000	2.50%	69.44%
B-2	709.11	852.79	859.28	10.52%	1570	1550	13200	9.39%	10.74%
P-1	111.54	120.95	113.14	4.37%	1250	1302	1240	2.54%	41.88%
P-2	12.23	14.92	13.33	10.03%	183	190	198	3.94%	60.72%
P-3	1.29	3.68	1.69	57.69%	21	13	23	27.85%	51.72%
P-4	0.95	1.40	1.08	20.25%	6.9	5.8	5.1	15.29%	24.49%
P-5	0.86	0.73	1.05	18.28%	5.5	4.5	6.3	16.60%	9.19%
P-6	0.43	0.66	0.90	34.88%	7.5	7.7	9.3	12.08%	65.37%

[a] Values reflect copies/μL based on serial dilutions of positive plasmid DNA standard.

[b] Values reflect copies/20μL ddPCR reaction.

[c]. Decreased CV% was calculated by 100%×(CV% $_{ddPCR}$-CV% $_{qPCR}$)/ CV% $_{qPCR}$.
